# MLK3 Is Associated With Poor Prognosis in Patients With Glioblastomas and Actin Cytoskeleton Remodeling in Glioblastoma Cells

**DOI:** 10.3389/fonc.2020.600762

**Published:** 2021-02-22

**Authors:** Yan Zhu, Jin-Min Sun, Zi-Chen Sun, Feng-Jiao Chen, Yong-Ping Wu, Xiao-Yu Hou

**Affiliations:** ^1^Jiangsu Key Laboratory of Brain Disease Bioinformation, Research Center for Biochemistry and Molecular Biology, Xuzhou Medical University, Xuzhou, China; ^2^State Key Laboratory of Natural Medicines, School of Life Science and Technology, China Pharmaceutical University, Nanjing, China; ^3^Laboratory of Clinical and Experimental Pathology, Department of Pathology, Xuzhou Medical University, Xuzhou, China

**Keywords:** actin cytoskeleton remodeling, epidermal growth factor receptor kinase substrate 8 (EPS8), glioblastoma prognosis, glioma progression, isocitrate dehydrogenase (IDH), mixed lineage kinase 3 (MLK3), primary and recurrent glioblastoma multiforme (GBM)

## Abstract

Mixed lineage kinase 3 (MLK3) has been implicated in human melanoma and breast cancers. However, the clinical significance of MLK3 in human gliomas and the underlying cellular and molecular mechanisms remain unclear. We found that MLK3 proteins were highly expressed in high-grade human glioma specimens and especially prevalent in primary and recurrent glioblastoma multiforme (GBM). High levels of MLK3 mRNA were correlated with poor prognosis in patients with isocitrate dehydrogenase (*IDH*)-wild-type (wt) gliomas. Furthermore, genetic ablation of MLK3 significantly suppressed the migration and invasion abilities of GBM cells and disrupted actin cytoskeleton organization. Importantly, MLK3 directly bound to epidermal growth factor receptor kinase substrate 8 (EPS8) and regulated the cellular location of EPS8, which is essential for actin cytoskeleton rearrangement. Overall, these findings provide evidence that MLK3 upregulation predicts progression and poor prognosis in human *IDH*-wt gliomas and suggest that MLK3 promotes the migration and invasion of GBM cells by remodeling the actin cytoskeleton *via* MLK3-EPS8 signaling.

## Introduction

Gliomas, one of the most prevalent forms of primary brain tumors, are classified into four grades (grade I–IV) in line with the World Health Organization (WHO) 2016 classification criteria (1). Glioblastoma multiforme (GBM) is a malignant grade IV tumor with poor prognosis. According to the statistics, the 3-year overall survival of patients with GBM is approximately 12% (2). Tumor invasion and immune evasion account for the main causes of recurrence and death in patients with GBM. Therefore, understanding the underlying cellular and molecular events may provide promising molecular markers for GBM diagnosis, prognosis, and targeted therapy.

Mixed lineage kinase 3 (MLK3), also known as mitogen-activated protein kinase kinase kinase 11 (MAP3K11) and Src-homology 3 (SH3) domain-containing proline-rich kinase (SPRK), is a member of the serine/threonine protein kinase family encoded by the *MAP3K11* gene in humans (3). MLK3 is involved in human melanoma and breast cancers (4–6). High levels of MLK3 mRNA are found in metastatic primary malignant melanoma tissues (4). Breast tumors express a comparable level of MLK3 proteins, whereas MLK3 kinase activity is profoundly reduced and inversely correlated with tumor grades in human epidermal growth factor receptor (EGFR) 2-positive breast cancer tissues (5, 6). In diverse human cancer cell lines, MLK3 is involved in multiple cellular processes, including proliferation, proapoptosis, migration, and invasion (4–11). MLK3-JNK signaling has been reported to be related to EGFR activation-driven migration and invasion of GBM cell line (10). However, the pathophysiological function of MLK3 in the progression and prognosis of human gliomas remains unknown, and how MLK3 promotes the development of gliomas has not been well understood.

Cancer cell migration and invasion involves integrated complexes and is a dynamic process that requires actin cytoskeletal rearrangement to change the cell shape and generate the driving force for cell movement. A group of regulatory molecules are involved in cytoskeletal remodeling, including EGFR kinase substrate 8 (EPS8) (12–15). EPS8 is responsible for actin cytoskeleton formation and facilitates the migratory and invasive capacities of GBM cells (16). Furthermore, recent findings implicate that actin cytoskeleton remodeling drives cancer cell resistance to antitumor immunity (17). Altogether, elucidating the roles of MLK3 in actin cytoskeleton regulation is essential for understanding glioma progression and invasion.

In this study, we examined the expression of MLK3 in human glioma tissue specimens. Additionally, we determined the correlation between MLK3 protein and mRNA levels and glioma progression and poorer prognosis in patients with GBM. Furthermore, we investigated whether and how MLK3 is involved in GBM cell migration, invasion, and actin cytoskeletal remodeling. Our data provide evidence that MLK3 is a valuable biomarker for predicting the prognosis and towards targeted therapy of GBM.

## Methods

### Human Tissue Analysis

Glioma tissues (WHO grade I, n = 6; grade II, n = 23; grade III, n = 20; and grade IV, n = 48) were obtained from the Department of Pathology of the Affiliated Hospital of Xuzhou Medical University between 2016 and 2017. All samples were identified by pathologists according to the 2016 WHO classification criteria.

Publicly available RNA-seq data of gliomas were collected from the Freije dataset (https://www.oncomine.org) and the CGGA database (https://www.cgga.org.cn). The Freije dataset includes 81 glioma tissues (WHO grade III, n = 24 and grade IV, n = 57), and the Chinese Glioma Genome Atlas (CGGA) dataset includes 325 glioma tissues. After incomplete data (grade, overall survival, isocitrate dehydrogenase (*IDH*) mutation status, 1p/19q codeletion status, O^6^-methylguanine-DNA methyltransferase (*MGMT*) promoter methylation status) were deleted, 286 glioma tissues in the CGGA dataset (WHO grade II, n = 86; grade III, n = 68; and grade IV, n = 132) were used to analyze overall survival and the levels of MLK3, EGFR, MAPK8, MAPK9, MAPK10, and EPS8 mRNA. The median levels of various mRNA in glioma samples were chosen as the respective cut-off points.

### Antibodies and Plasmids

Rabbit polyclonal anti-MLK3 (#sc-536) and mouse monoclonal anti-EPS8 (#sc-390257) antibodies were obtained from Santa Cruz Biotechnology. Mouse monoclonal anti-GAPDH (glyceraldehyde phosphate dehydrogenase) (#60004-1-Ig) antibody and rabbit polyclonal anti-vinculin antibody (#26520-1-AP) were obtained from Proteintech. Mouse monoclonal anti-Myc (#05-419) and rabbit polyclonal anti-GST (glutathione S-transferase) (#06-332) antibodies were obtained from Millipore. Horseradish peroxidase-conjugated goat anti-mouse IgG (#A28177) and goat anti-rabbit IgG (#31460), goat anti-mouse IgG-Alexa Fluor 488 (#A-11029), and goat anti-rabbit IgG-Alexa Fluor 594 (#A11037) were obtained from Invitrogen. Phalloidin (#PHDR1) was obtained from Cytoskeleton.

Full-length human MLK3 cDNA (puc19-hMAP3K11, #HG11067-U) was obtained from Sino Biological Inc. and subcloned into pcDNA3.1-Myc plasmids. EPS8 cDNA was amplified from human HEB cells and cloned into the pcDNA3.1-His plasmids. Human cDNA coding for MLK3 (1–104 aa) and MLK3 (632–847 aa) were amplified and cloned into pGEX-4T-1 plasmids. All recombinant plasmids were identified by sequencing.

### Immunohistochemical Staining

Paraffin-embedded glioma tissue sections were deparaffinized and hydrated using xylene and graded alcohols. Antigen retrieval was performed with high pressure for 3 min. The tissues were blocked with 3% bovine serum albumin for 20 min at room temperature. Primary antibodies were incubated overnight at 4°C. Then, biotinylated secondary antibodies were incubated for 30 min at room temperature. The staining was carried out using an ABC reagent kit (VECTASTAIN) and a 3,3-diaminobenzidine peroxidase substrate reagent kit (Vector, #SK-4100). The nuclei were treated with hematoxylin staining, and the sections were mounted on glass slides. Images were acquired with Nikon microscopy. The evaluation of MLK3 staining was as previously described (18). According to staining intensity and area, MLK3 staining was categorized into scores 0–12. The median level of MLK3 (score 6) as the cut-off point, the samples were divided into low/high expression of MLK3 groups.

### Cell Culture and Transfection

The human GBM cell lines U87, U118, U251, U343, and T98G were maintained in Dulbecco’s modification of Eagle’s medium (DMEM, Gibco, #12000-014) supplemented with 10% fetal bovine serum (FBS). Cells were grown at 37°C and 5% CO_2_. All cell lines were authenticated through short tandem repeat DNA fingerprinting from Cell Bank/Stem Cell Bank, The Committee of Type Culture Collection of Chinese Academy of Sciences (Shanghai, China) in August 2018. Plasmid transfections were carried out with Lipofectamine 3000 (Invitrogen, #L3000-015).

### Knockout of the *MAP3K11* Gene

The knockout of the *MAP3K11* gene was performed by the CRISPR/Cas9 system. The special guide (sg) RNA1 sequence (5’-CACTGGGCTCGTAGTCGAAC-3’) and sgRNA2 sequence (5’-TTGAGTCCTCCAGACGTCGG-3’) targeting exons 1 and 7, respectively, were cloned into pSpCas9 (BB)-2A-Puro (PX459) vector. PX459 recombinants vector were transfected into U118 and U251 cells. Single cell colonies were screened with 0.75 μg/ml puromycin and identified by sequencing and western blot assays. The fragments of genomic DNA were amplified with forward primer F1 (5’-AAAAAGACCCAACCGGAGT-3’) and reverse primers R1 (5’-CAGCCTTGAGGGCAATGAT-3’) and R2 (5’-AGAGCAACCAGGGCAGGAC-3’). The PCR products were sequenced.

### Transwell Migration and Invasion Assays

Cells were treated with serum-free DMEM for 14 h. Then, cells (5×10^4^) were suspended in serum-free DMEM and added to the upper chamber of a 24-well transwell plate (Corning, #3422). For the invasion assay, the upper chambers were precoated with a Matrigel Basement Membrane Matrix (BD Biosciences, #356234). DMEM supplemented with 10% FBS was added to the lower chambers. The cells were cultured at 37°C for 10 h ~ 24 h. The chambers were washed with phosphate-buffered saline (PBS), fixed with 4% paraformaldehyde for 20 min and washed with PBS. The cells on the upper surface of the membrane were removed with a cotton swab. The cells on the bottom surface were stained with Giemsa staining.

### Immunofluorescence Analysis

Cells were plated on glass slides, washed with PBS, fixed with 4% paraformaldehyde for 10 min at 4°C, permeabilized with Triton X-100 (0.2%) for 15 min at room temperature, and blocked with 10% normal goat serum for 2 h at room temperature. Primary antibodies were incubated overnight at 4°C. The fluorescent secondary antibodies were incubated for 2 h at room temperature. The nuclei were stained with 4-6-diamidino-2-phenylindole (DAPI) (Sigma, #D8417). Coverslips were mounted on the glass slides, and images were taken with confocal microscopy.

### Immunoblot Analysis

Cell total protein was separated by 10% sodium dodecyl sulfate polyacrylamide gel electrophoresis (SDS-PAGE) and transferred to nitrocellulose membranes. After blocking with 3% bovine serum albumin for 2 h, the membranes were incubated with the primary antibody overnight, followed by horseradish peroxidase-conjugated secondary antibody for 1 h at 4°C. The specific proteins were detected by enhanced chemiluminescence reagent. The band intensity was quantified with ImageJ software.

### Immunoprecipitation (IP)

U251 cells were transfected with pcDNA3.1-Myc-MLK3 and pcDNA3.1-His-EPS8 plasmids for 24 h, and protein lysates were obtained. IP assay of MLK3-EPS8 interaction was performed with anti-EPS8, anti-MLK3 or anti-Myc antibodies as previously described (19).

### GST Pull-Down Assay

GST-fused MLK3 (1–104 aa) and MLK3 (632–847 aa) proteins were expressed in BL21 cells and purified with the Pierce™ GST Protein Interaction Pull-Down Kit (Thermo, #21516) according to the manufacturer’s instructions. The purified proteins were incubated with the protein lysates from pcDNA3.1-EPS8-transfected HEK293 cells for 2 h at 4°C. The beads were washed and boiled in loading buffer for the immunoblot assay.

### Statistical Analysis

All data are presented as the mean ± standard deviation (SD). Statistical analyses were performed using GraphPad Prism 7.0 software. Pearson correlation analysis and chi-square (*X^2^*) tests were conducted to detect correlations. The Kaplan-Meier method was applied to assess overall survival. For parametric data, two-tailed Student’s *t*-tests and one-way ANOVA were used to examine differences. For non-parametric data, a two-sided Mann-Whitney test was used. *P <* 0.05 was considered statistically significant.

## Results

### Mixed Lineage Kinase 3 Is Highly Expressed in High-Grade Human Glioma Specimens

**Figure 1:**
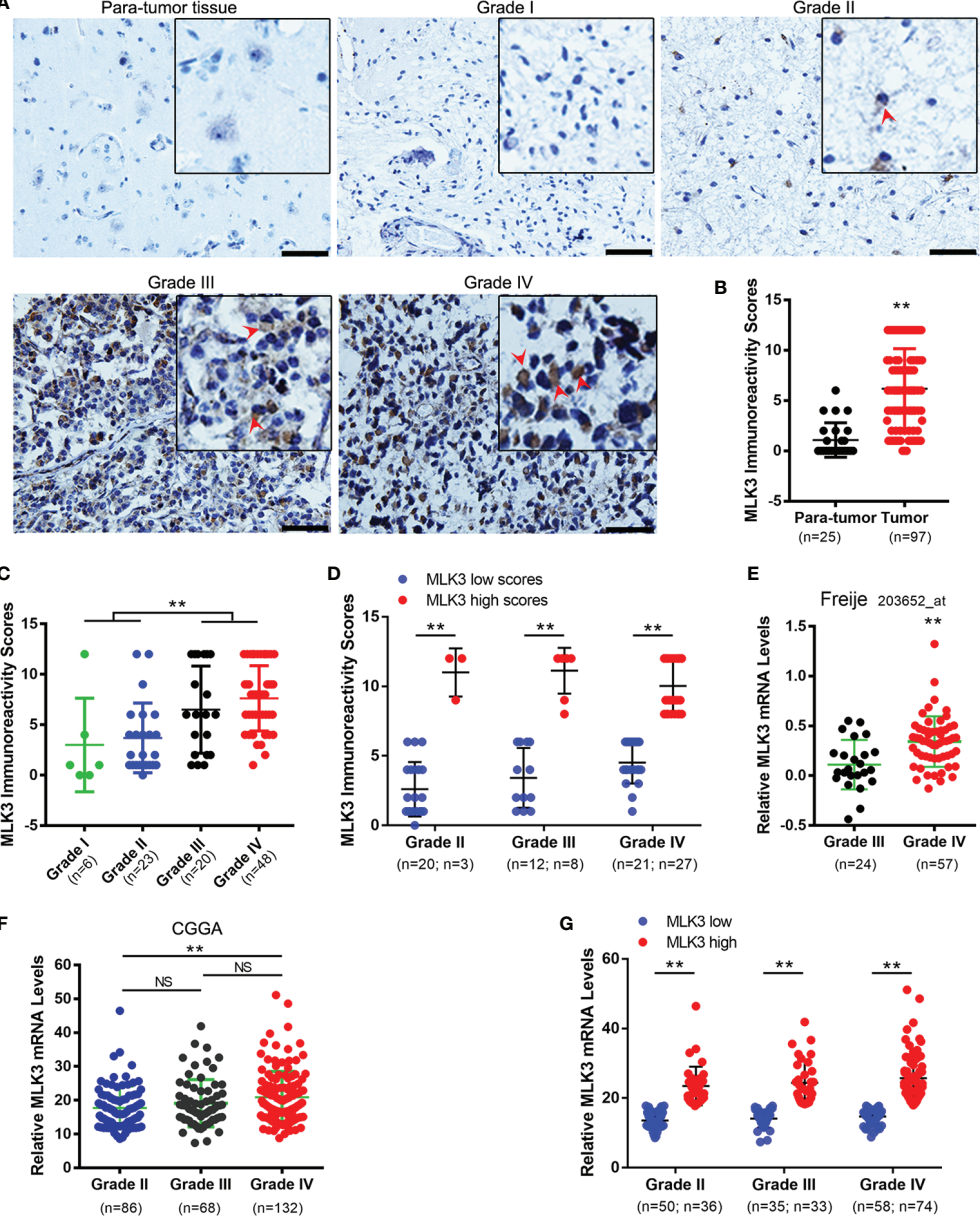
MLK3 is highly expressed in high-grade human glioma specimens. **(A–C)** MLK3 protein levels are upregulated in high-grade gliomas. **(A)** Representative immunohistochemistry IHC images of MLK3 in human glioma tissues. Scale bars, 50 μm. **(B)** MLK3 expression in para-tumor tissues (n = 25) and glioma tissues (n = 97). **(C)** Comparison of MLK3 expression between low-grade (grade I and II, n = 29) and high-grade (grade III and IV, n = 68) glioma tissues. Mann-Whitney *U* test. **P < 0.01. **(D)** The variation of MLK3 levels in the same grade gliomas. Mann-Whitney *U* test, **P < 0.01. **(E, F)** MLK3 mRNA levels are upregulated in high-grade gliomas. mRNA sequencing-based glioma datasets from Oncomine database **(E)** and the CGGA database **(F)** were analyzed by two-tailed Student’s *t*-tests and one-way ANOVA. NS, not significant. **P < 0.01. **(G)** The variation of MLK3 mRNA levels in the same grade gliomas. Two-tailed Student’s *t*-tests, **P < 0.01.

First, 97 clinical glioma tissue specimens were analyzed with immunohistochemistry (IHC) to determine the protein expression and subcellular distribution of MLK3. As shown in **Figures 1A, B**, the expression levels of MLK3 protein were higher in glioma tissues than in para-tumor tissues. Clearly, MLK3 proteins were mainly localized in the cytoplasm of glioma cells. Next, we assessed the correlation between MLK3 levels and clinicopathological characteristics (tumor size and WHO grade). IHC analysis showed that the levels of MLK3 protein were closely correlated with age and tumor grade (**Table 1**). MLK3 staining was weaker in low-grade glioma tissues (grade I and II) than in high-grade glioma tissues (grade III and IV) (**Figures 1A, C**). In addition, there was significant variation of MLK3 levels among patients within the same grade gliomas (**Figure 1D**). The percentage of high MLK3 level cases increased with the grade of glioma.

**Table 1:** MLK3 staining and clinicopathological characteristics of 97 glioma patients.

Variables		Number	MLK3 expression		*X^2^* value	*P* value
			High (%)	Low (%)		
**Age**	≤50	44	20 (45.5)	24 (54.5)	4.149	0.042^*^
	>50	53	35 (66.0)	18 (34.0)		
**Gender**	Male	60	36 (60.0)	24 (40.0)	0.697	0.404
	Female	37	19 (51.4)	18 (48.6)		
**Tumor size**	<5 cm	31	17 (54.8)	14 (45.2)	0.000	1.000
	≥5 cm	31	17 (54.8)	14 (45.2)		
**WHO grade**	Low (I+II)	29	7 (24.1)	22 (75.9)	17.867	0.000^**^
	High(III+IV)	68	48 (70.6)	20 (29.4)		

*P < 0.05, **P < 0.01.

Next, we explored MLK3 mRNA levels in glioma tissues by using RNA sequencing (RNA-seq) data from the Chinese Glioma Genome Atlas (CGGA) database and the Freije dataset from the Oncomine database. The results showed that the levels of MLK3 mRNA were significantly increased in high-grade gliomas (grade IV) and closely associated with the progression of gliomas (**Figures 1E, F**). MLK3 mRNA levels showed significant variation within the same grade (**Figure 1G**), which is consistent with the results of IHC analysis.

These findings suggest that MLK3 is significantly upregulated in high-grade human glioma tissues and positively associated with a malignant phenotype.

### High Mixed Lineage Kinase 3 Levels Are Prevalent in Isocitrate Dehydrogenase Gene-Wild-Type Glioblastoma Multiforme and Correlated With the Poor Prognosis of Patients

**Figure 2:**
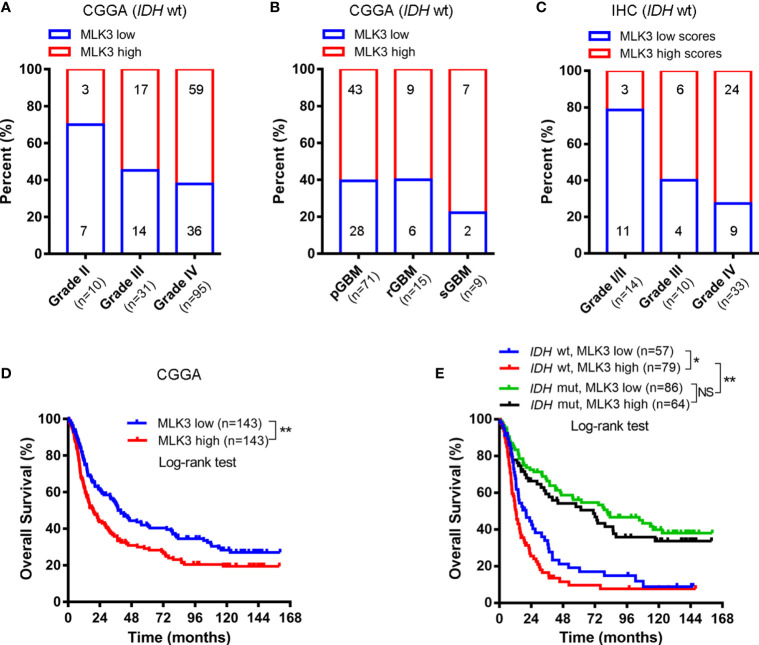
High MLK3 levels are correlated with poor prognosis in patients with *IDH*-wt GBM. **(A, B)** High levels of MLK3 mRNA are prevalent in *IDH*-wt GBM. The frequencies of high MLK3 mRNA levels in *IDH*-wt gliomas (n = 136), primary GBM (n = 71), recurrent GBM (n = 15), and secondary GBM (n = 9) were analyzed. The data were derived from the CGGA dataset. The median level of MLK3 mRNA in glioma samples was chosen as the cut-off point. pGBM, primary GBM; rGBM, recurrent GBM; sGBM, secondary GBM. **(C)** MLK3 abundantly expressed in *IDH*-wt GBM. The percent of high MLK3 protein levels in *IDH*-wt gliomas (n = 57) was investigated. The data were obtained from IHC analysis. **(D, E)** High levels of MLK3 mRNA are associated with the poor prognosis of patients. Kaplan-Meier survival analyses for MLK3 mRNA expression (n = 286) **(D)** and MLK3 mRNA expression and *IDH* gene status (n = 286) **(E)**. The data were derived from the CGGA dataset. Log-rank test. NS, not significant. *P < 0.05, **P < 0.01.

The detection of several molecular markers, including *IDH1* mutation, 1p/19q codeletion, *MGMT* promoter methylation status, and *EGFR* amplification has been applied with clinical diagnoses of gliomas (1). We analyzed the correlation between MLK3 levels and the status of these biomarkers by using CGGA data and IHC analysis. The results revealed that the expression of MLK3 mRNA in human gliomas was correlated with *IDH* status but not 1p/19q codeletion and *MGMT* promoter methylation status. While it was negatively related to EGFR mRNA levels (**Table 2**). We also analyzed the correlation between MLK3 levels and downstream regulators. The results showed that MLK3 mRNA in human gliomas was negatively related to MAPK8, MAPK9, and MAPK10 (**Table 2**). Additionally, MLK3 was highly expressed in *IDH*-wt gliomas and especially prevalent in *IDH*-wt GBM (59/95) (**Figure 2A**). In *IDH*-wt GBM, high levels of MLK3 frequently occurred in primary GBM (43/71), recurrent GBM (9/15) and secondary GBM (7/9) (**Figure 2B**). The results of the IHC analysis confirmed that the MLK3 protein was abundantly expressed in *IDH*-wt GBM (24/33) (**Figure 2C**) and are in accordance with the results of the bioinformatics analysis.

**Table 2:** Relationship between MLK3 mRNA expression and clinicopathologic variables of patients with gliomas.

Variables		Number	MLK3 expression		*X^2^* value	*P* value
			High (%)	Low (%)		
***IDH***	Wildtype	136	79 (58.1)	57 (41.9)	6.785	0.009^**^
	Mutation	150	64 (42.7)	86 (57.3)		
**1p/19q codeletion**	Codel	57	24 (42.1)	33 (57.9)	1.775	0.183
	Non-codel	229	119 (52.0)	110 (48.0)		
***MGMT* promoter**	Methylated	147	73 (49.7)	74 (50.3)	0.014	0.906
	Unmethylated	139	70 (50.4)	69 (49.6)		
***EGFR***	High	143	58 (40.6)	85 (59.4)	10.196	0.001^**^
	Low	143	85 (59.4)	58 (40.6)		
***MAPK8***	High	143	45 (31.5)	98 (68.5)	39.287	0.000^**^
	Low	143	98 (68.5)	45 (31.5)		
***MAPK9***	High	143	53 (37.1)	90 (62.9)	19.147	0.000^**^
	Low	143	90 (62.9)	53 (37.1)		
***MAPK10***	High	143	56 (39.2)	87 (60.8)	13.441	0.000^**^
	Low	143	87 (60.8)	56 (39.2)		

** P < 0.01.

We further studied the correlation between MLK3 expression and the overall survival of patients with gliomas by bioinformatics analysis. The Kaplan-Meier analysis showed that high levels of MLK3 were associated with the poor prognosis of patients (**Figure 2D**). Glioma patients with the *IDH*-mutant (mut) phenotype had a better prognosis than patients with the *IDH*-wt phenotype. Remarkably, in *IDH*-wt gliomas, patients with comparatively lower levels of MLK3 had a higher overall survival rate than patients with high levels of MLK3 (**Figure 2E**). By contrast, there was no difference in the overall survival of *IDH*-mut patients with either high or low levels of MLK3 (**Figure 2E**).

These data suggest that MLK3 upregulation predicts poorer prognosis in *IDH*-wt gliomas.

### Mixed Lineage Kinase 3 Promotes Glioblastoma Multiforme Cell Migration and Invasion and Is Required for Actin Cytoskeleton Rearrangement

**Figure 3:**
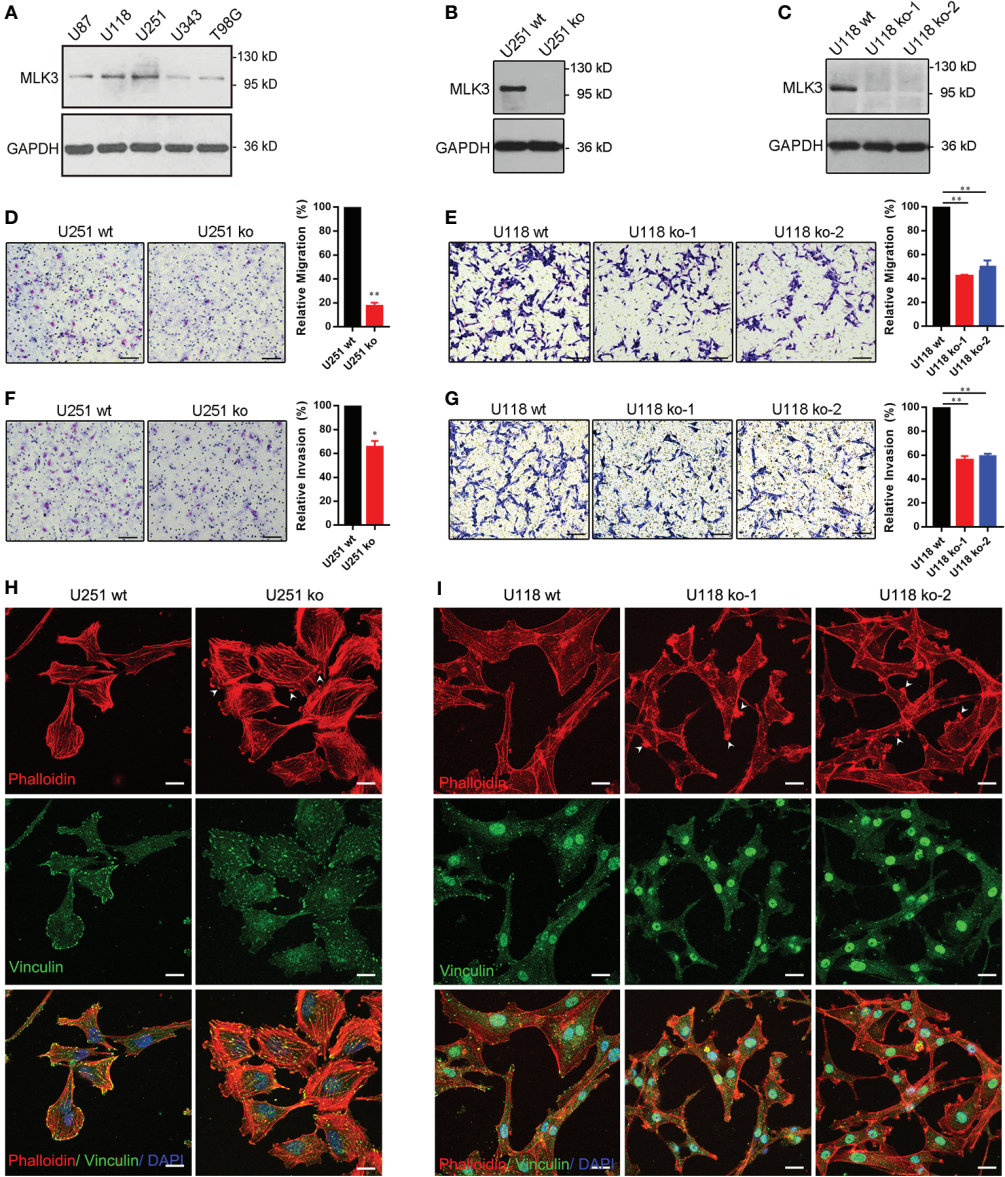
MLK3 deficiency attenuates glioblastoma multiforme (GBM) cell migration and invasion and disrupts actin cytoskeleton rearrangement. **(A)** MLK3 proteins are overexpressed in highly migrating GBM cells. Cells were treated with serum-free DMEM for 48 h. Immunoblot analysis of MLK3 levels in several GBM cell lines. GAPDH was used as a loading control. **(B, C)** Immunoblot assay of the *MAP3K11* gene knockout in U251 **(B)** and U118 **(C)** cells. GAPDH was used as a loading control. **(D–G)** Transwell assay of GBM cell migration and invasion in *MAP3K11* gene knockout U251 **(D, F)** and U118 **(E, G)** cells. Two-tailed Student’s *t*-test. Scale bars, 100 μm; n = 3; **P < 0.01. **(H, I)** The loss of MLK3 disrupts actin cytoskeleton rearrangement. The results shown are representative of at least three independent cultures. Phalloidin was used to label F-actin (red), anti-vinculin antibody was used to stain focal adhesion structure (green), and DAPI was used to stain nuclei (blue). White arrows indicate the actin based “mass-like structures.” Scale bars, 20 μm.

To assess the roles of MLK3 in the biological behaviors of glioma cells, we first detected the protein levels of MLK3 in high-migration glioma cell lines (U87, U118, and U251) and low-migration glioma cell lines (U343 and T98G) 48 h after serum starvation (**Figure 3A**). MLK3 was highly expressed in U87, U118, and U251 cells, implying that MLK3 could be related to the migration and invasion of GBM cells. To evaluate the roles of MLK3 in the migration and invasion of GBM cells, we generated *MAP3K11* gene knockout U251 and U118 cells by the CRISPR/Cas9 system. Positive clones (U251 ko, U118 ko-1, and U118 ko-2) were confirmed by sequencing and immunoblot analysis (**Supplementary Figure S1**, **Figures 3B, C**). The loss of MLK3 robustly restrained cell migration in U251 and U118 cells compared to wt cells (**Figures 3D, E**). The Matrigel assay showed that MLK3 ablation evidently suppressed U251 and U118 cell invasion (**Figures 3F, G**). The above results suggest that MLK3 is required for the migration and invasion of GBM cells *in vitro*.

Next, the MLK3 kinase inhibitors CEP-701 was used to test the roles of MLK3 activity in the regulation of GBM cell migration and invasion. Immunoblot analysis showed that CEP-701 effectively inhibited the activity of MLK3 (**Supplementary Figure S2A**). A wound healing assay showed that CEP-701 (at the appropriate concentration) significantly inhibited the wound closure of U87, U251, and T98G cells in comparison to vehicle-treated cells (**Supplementary Figures S2B–D**). Transwell assays showed that CEP-701 (400 nM) markedly suppressed the migration of U251 and U87 cells (**Supplementary Figures S2E, F**). The Matrigel assay revealed that the invasion ability of U251 and U87 cells was remarkably reduced after treatment with CEP-701 (400 nM) (**Supplementary Figures S2G, H**).

Actin cytoskeleton remodeling is a vital event in cell migration and invasion. To clarify the role of MLK3 in actin cytoskeleton organization, F-actin and vinculin (for focal adhesion) staining were carried out to identify changes in the actin cytoskeleton. The results showed that MLK3 ablation in U251 and U118 cells resulted in marked changes of the actin cytoskeleton, and actin based “mass-like structures” appeared on the edge of cells (**Figures 3H, I**). MLK3 ablation increased stress fibers in U251 cells (**Figure 3H**), while the distribution of vinculin obviously decreased on the edge of U251 ko and U118 ko cells (**Figures 3H, I**). More interestingly, the altered cell morphology and more filamentous protuberances were observed by ectopic expression of MLK3 in T98G cells (**Supplementary Figure S3**). These findings indicate that MLK3 contributes to actin cytoskeleton remodeling.

### Mixed Lineage Kinase 3 Directly Binds to Epidermal Growth Factor Receptor Kinase Substrate 8

**Figure 4:**
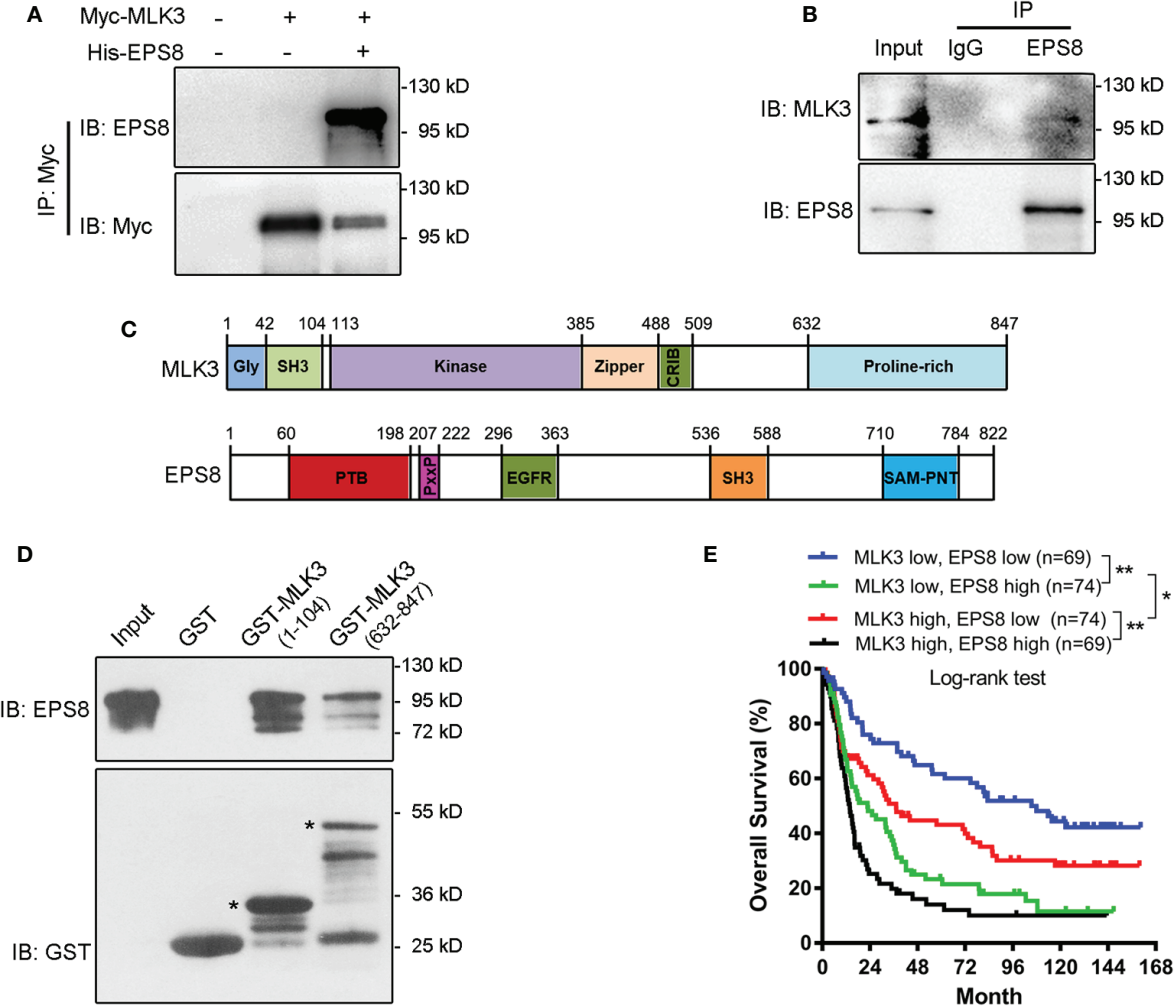
MLK3 directly binds with EPS8. The results shown are representative of at least three independent experiments. **(A, B)** Overexpressed and endogenous MLK3 binds to EPS8 in U251 cells. U251 cells were cotransfected with pcDNA3.1-Myc-MLK3 and pcDNA3.1-His-EPS8 plasmids for 24 h, with the pcDNA3.1 plasmid as a control. The lysates were immunoprecipitated using anti-Myc **(A)**, anti-EPS8 **(B)** or control IgG, EPS8, or MLK3 was detected with an immunoblot assay. **(C)** The molecular structure of MLK3 and EPS8. **(D)** AAs 1–104 and 632–847 of MLK3 directly bound to EPS8 *in vitro*. GST-tagged MLK3 segments were expressed in BL21 cells. The purified proteins were incubated with the protein lysates from pcDNA3.1-EPS8-transfected HEK293 cells. The proteins were immunoblotted with indicated antibodies. * the target protein. **(E)** The expression of MLK3 and EPS8 predicts the overall survival of patients. The data were derived from the CGGA dataset (n = 286). Kaplan-Meier survival analyses for MLK3 and EPS8 mRNA levels. Log-rank test. *P < 0.05, **P < 0.01.

Previous studies have indicated that EPS8 controls actin cytoskeleton reorganization (20, 21). To elucidate the molecular mechanism underlying MLK3-mediated cell migration and invasion, we identified the molecular interactions of MLK3 and EPS8. Co-IP analysis showed that overexpressed (**Figure 4A**) and endogenous MLK3 (**Figure 4B**) interacted with EPS8 in U251 cells. Furthermore, *in vitro* GST pull-down analysis showed that the c-terminal domain of MLK3 (1–104 aa) and the proline-rich domain (PRD) of MLK3 (632–847 aa) directly bound to EPS8 (**Figure 4D**). In addition, MLK3 cooperates with EPS8 and affects the overall survival of patients with gliomas. Bioinformatics analysis showed that high levels of MLK3 and EPS8 in gliomas are correlated with a significantly worse overall survival in patients (**Figure 4E**). These results suggest that MLK3-EPS8 signaling may be involved in the development of gliomas.

### Mixed Lineage Kinase 3 Regulates the Localization of Epidermal Growth Factor Receptor Kinase Substrate 8

**Figure 5:**
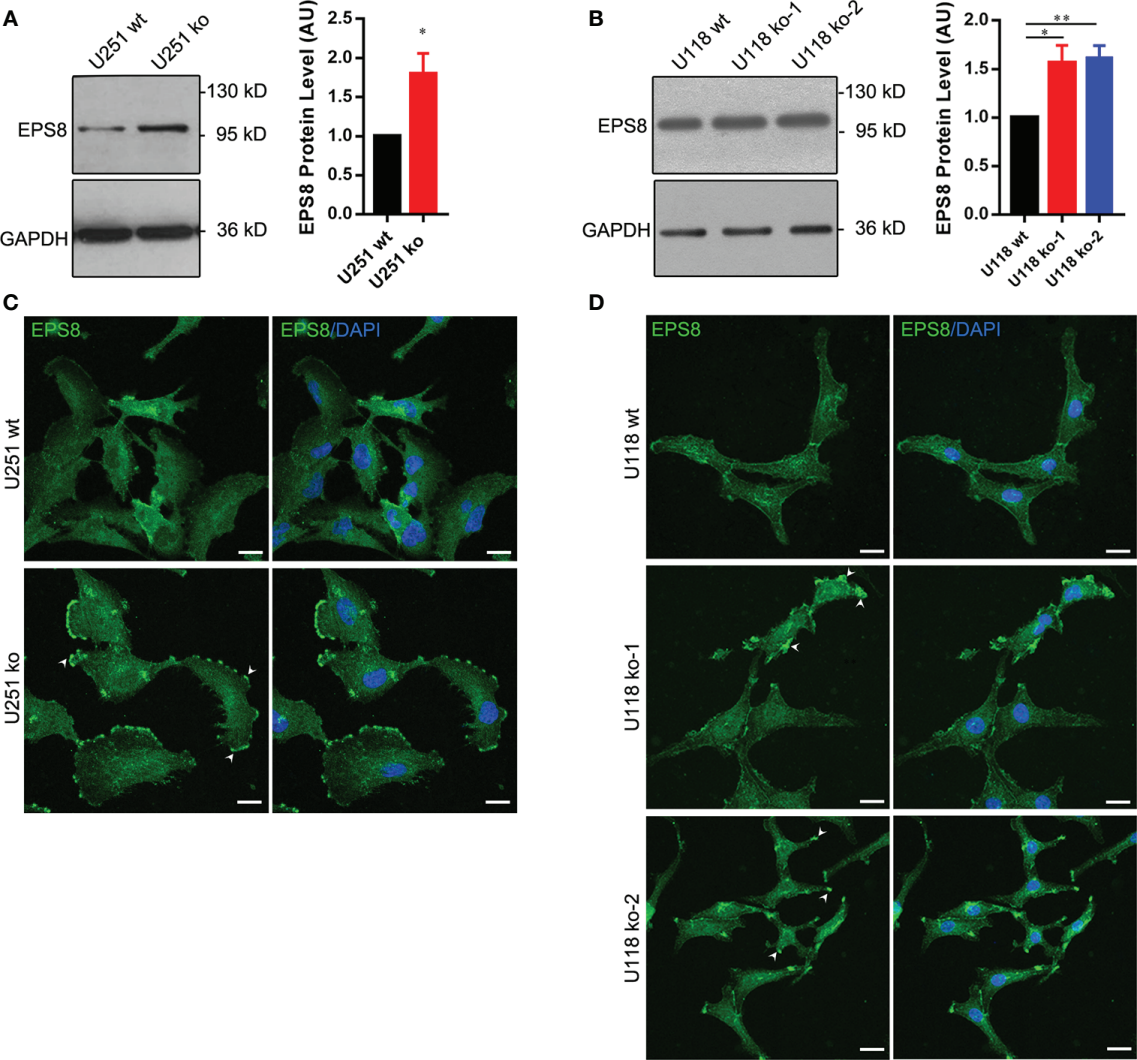
MLK3 regulates the localization of EPS8 in U251 and U118 cells. **(A, B)** The *MAP3K11* gene knockout increases the expression of EPS8 in U251 **(A)** and U118 **(B)** cells. Relative levels of MLK3 were normalized to U251 wt or U118 wt groups. GAPDH was used as a loading control. Two-tailed Student’s *t*-test. n = 3, *P < 0.05, **P < 0.01. AU, arbitrary unit. **(C, D)** The *MAP3K11* gene knockout alters the location of EPS8 in U251 **(C)** and U118 **(D)** cells. The results shown are representative of at least three independent cultures. EPS8 (green); DAPI-stained nuclei (blue). Scale bars, 20 μm.

To further explore the mechanisms of MLK3-mediated actin cytoskeleton remodeling, we examined the localization and expression of EPS8 in MLK3-depleted U118 and U251 cells. Immunoblot assays showed that EPS8 was upregulated in MLK3-depleted U118 and U251 cells (**Figures 5A, B**). Additionally, the immunofluorescence assay results showed that the subcellular localization of EPS8 was disrupted in MLK3-ablated cells, and mass-like structures were assembled on the cell edges (**Figures 5C, D**). These data suggest that MLK3 is critical for actin cytoskeleton rearrangement by regulating EPS8 localization in GBM cells.

## Discussion

GBMs are devastating malignancies with highly migratory and invasive behavior. Despite advancements in clinical diagnosis and treatment, recurrence remains universal, and available salvage strategies are rare. Therefore, identifying special molecular markers for the early diagnosis, prognosis evaluation, and targeted therapy of GBM is of great significance. Our findings provide the first evidence that MLK3 upregulation plays an essential role in tumor progression and correlates with an unfavorable overall survival in patients with *IDH*-wt GBM. MLK3 may serve as a valuable diagnostic and prognostic marker and may be a promising therapeutic target for GBM therapy.

To date, patients with *IDH*-wt gliomas exhibit the poorest outcomes, and few targeted agents are therapeutically effective for *IDH*-wt gliomas. The present study provides evidence that MLK3 might be required for the evaluation of prognosis and targeted therapy of *IDH*-wt gliomas. Previous studies indicated that the *IDH* and *IDH2* mutations are more frequent in grade II–III gliomas and secondary GBMs (22–24). Approximately 80% of secondary GBMs have somatic mutations in *IDH*, which are absent in primary GBM (25). Patients with *IDH* mutations have a better prognosis, which is consistent with our findings. An increase in R (-)-2-hydroxyglutarate in gliomas harboring *IDH* mutations has been found to prevent the association of MLK3 with Cdc42 and further inhibit the activity of MLK3, which may be one of the reasons for the better prognosis in patients with *IDH*-mut gliomas (26, 27). More notably, our findings showed that *IDH*-mut gliomas present with low levels of MLK3, suggesting that mutant *IDH* not only blocks MLK3 activity but also reduces MLK3 expression. Although there is no correlation between the MLK3 levels and overall survival of patients with *IDH*-mut gliomas, high levels of MLK3 are positively correlated with poor prognosis in *IDH-*wt gliomas. Therefore, the prognosis of patients with *IDH-*wt gliomas can be reasonably evaluated by MLK3. In addition, this study demonstrated that MLK3 cooperates with EPS8, which might aid in prognosis prediction for patients with gliomas.

MLK3 appears to function as an oncogene to promote cell migration and invasion through the MLK3/JNK pathway in human breast and gastric cancer cells (7, 8, 10), the MLK3/FRA-1/MMP-1 axis in human triple-negative breast cancer cells (28), or MLK3/ERK signaling in ovarian and colorectal cancer cells (9, 11). Here, our data showed that MLK3 is responsible for the migration and invasion abilities of GBM cells *via* MLK3/EPS8 signaling. MLK3 has been reported to contribute to EGF-induced GBM cell migration and invasion (10). Here, we found that MLK3 mRNA levels in human gliomas was negatively related to EGFR and MAPK8, MAPK9, MAPK10 mRNAs levels, and MLK3 downregulation reduces GBM cell migration and invasion without EGF induction, suggesting that the MLK3 regulation of cell migration and invasion is EGF signaling-independent.

Cell migration is a multipurpose process (29, 30) and drives the progression of cancer (31, 32). The alteration of the actin cytoskeleton is critical in the process of cell migration and generates the forces that push cell migration. MLK3 silencing enhances stress fibers in breast cancer cells (33). In this study, we confirmed that genetic depletion of MLK3 disturbs actin cytoskeleton organization. However, the molecular mechanisms underlying actin cytoskeleton remodeling remain unclear. Previous studies showed that EPS8 directly binds to actin and blocks actin prolongation by its effector domain (648–821 aa) *via* its capping activity to activate Rac signaling (34, 35). Therefore, the accurate localization of EPS8 is essential and contributes to actin rearrangement. In this study, we found that MLK3 interacts with EPS8 and controls the localization of EPS8. Following the loss of MLK3 in U118 and U251 cells, EPS8 expression was apparently increased, and its location was disarranged, which resulted in a number of mass-like structures on the cell edge. We verified that the regions of MLK3 (1–104 aa) with an SH3 domain and MLK3 (632–847 aa) containing PRD directly bind with EPS8. Targeting the two domains of MLK3 could be used as a valuable strategy for developing drugs for glioma therapy. Taken together, our results confirmed a novel mechanism by which MLK3 promotes cell migration and invasion *via* MLK3/EPS8 signaling.

The actin cytoskeleton is a dynamic cell structure that is attributed to diverse cellular processes, including cell morphogenesis, membrane trafficking, cell division, and immune response (17, 36–38). In addition, actin cytoskeleton dynamic remodeling has emerged as a critical event for glioma invasion. Therefore, controlling the change in actin is a feasible approach for glioma therapy. Currently, immunological therapies are crucial strategies for glioma therapy. Thus, tumor cells that escape from immunological surveillance are a barrier to effective immunotherapies. A study confirmed that the alteration of actin dynamics in tumor cells facilitates immune evasion (17). It would be interesting to ascertain whether MLK3 is involved in immune evasion of gliomas by regulating actin dynamics.

In summary, our findings have illustrated that as an oncogene, MLK3 may be a crucial regulator of the progression of gliomas and is associated with poor prognosis. As a consequence, the deletion of MLK3 in GBM cells decrease the capacity for cell migration and invasion and disrupt actin cytoskeleton remodeling. Furthermore, we provided a novel mechanism by which MLK3 facilitates glioma cell migration by regulating actin skeleton remodeling *via* MLK3/EPS8 signaling. Therefore, MLK3 is a potential target for therapy.

## Data Availability Statement

The original contributions presented in the study are included in the article/**Supplementary Material**, further inquiries can be directed to the corresponding author.

## Ethics Statement

In this study, human glioma tissue samples were approved for use by the Ethics Committee of the Affiliated Hospital of Xuzhou Medical University (No. XYFY2018-KL056-01). All samples were obtained and analyzed previously by pathologists according to the 2016 WHO classification criteria. Written informed consent has been provided by the participants' legal guardian/next of kin prior to the surgery. For re-use of these samples in this study, written informed consent was not required in accordance with the national legislation and the institutional requirements.

## Author Contributions

YZ and X-YH designed the study. J-MS and Y-PW designed and performed the staining and scoring protocols for the human glioma tissues. YZ and Z-CS performed the cellular and molecular experiments and analyzed the data. F-JC assisted with the gene knockout by CRISPR/Cas9, and YZ and X-YH wrote the manuscript. All authors contributed to the article and approved the submitted version.

## Funding

This work was supported by grants from the National Natural Science Foundation of China (81473185, 81673418), the Natural Science Foundation of the Jiangsu Higher Education Institutions of China (18KJA310007), a project funded by the Priority Academic Program Development of Jiangsu Higher Education Institutions (PAPD), and a project funded by the Jiangsu 333 Program (BRA2018059).

## Conflict of Interest

The authors declare that the research was conducted in the absence of any commercial or financial relationships that could be construed as a potential conflict of interest.
