# Supplementary material for: MLK3 Is Associated With Poor Prognosis in Patients With Glioblastomas and Actin Cytoskeleton Remodeling in Glioblastoma Cells
Source: Front Oncol. 2021 Feb 22;10:600762. doi: 10.3389/fonc.2020.600762 (PMC7937953; doi:10.3389/fonc.2020.600762)
Supplement: Supplementary file 1 [file DataSheet_1.docx]

**Supplementary material**

**MLK3 is associated with poor prognosis in patients with glioblastomas and actin cytoskeleton remodeling in glioblastoma cells**

Yan Zhu^1,2^, Jin-Min Sun^1,2.3^, Zi-Chen Sun^1^, Feng-Jiao Chen^1^, Yong-Ping Wu^3^, Xiao-Yu Hou^1,2,*^

**Materials and methods**

**Plasmids**

The MLK3 lentivirus expression vectors (pSLenti-CMV-MAP3K11-3×FLAG-PGK-Puro-WPRE) and control (pSLenti-CMV-MCS-3×FLAG-PGK-Puro) were obtained from Obio technology (Shanghai) Corp., Ltd. T98G cells stably expression MLK3 or control were screened with puromycine (1μg/mL).

**Wound healing assay**

Cells (3×10^5^) were seeded in 12-well plates. After 90% confluency was reached, the cell monolayers were scratched with a white pipette tip and washed three times using phosphate-buffered saline (PBS). The cells were treated for 12~48 h with serum-free DMEM containing CEP-701 (Sigma, #C7869) or vehicle according to the indicated conditions. The wound width was imaged on a Nikon microscope and quantified with ImageJ software (National Institutes of Health). Each well was recorded at six different parts of the wound. The wound width was measured at five equally distributed areas and calculated as the average width.

**Figure legends**

**Figure S1.** Generation of the *MAP3K11* gene knockout in U118 and U251 cells by the CRISPR/Cas9 system. A. Schematic location of sgRNA1 and sgRNA2 targeting the exon 1 and exon 7 regions of *MAP3K11* human genomic DNA, respectively (upper). The PAM sequences are shown in red. Schematic location of primers (lower). Primers (F1, R1, R2) were designed according to the location of sgRNAs. F1 (113-131 bp), R1 (1019-1037 bp), and R2 (8581-8599 bp). B, C. Detection of *MAP3K11* gene knockout clones by PCR amplification. Specific fragments were analyzed by agarose gel electrophoresis.

**Figure S2.** MLK3 inhibitors CEP-701prevents the migration and invasion of GBM cells. A. CEP-701 is an effective inhibitor of MLK3. U87 cells were treated with CEP-701 (50~800 nM) for 48 h. Immunoblot analysis of p-SAPK/JNK levels to evaluate the activity of MLK3. GAPDH was used as a loading control. B-D. Wound healing assay of GBM cell migration. U87 (B), U251 (C) and T98G (D) cells were treated with CEP-701 or vehicle for different times. NS, not significant. Scale bars, 500 μm; n = 6; **P* < 0.05, ***P* < 0.01. E-H. Transwell assay of GBM cell migration (E, F) and invasion (G, H). U251 and U87 cells were treated with CEP-701 (400 nM) or vehicle for 24 h. Two-tailed Student’s *t*-test. Scale bars, 100 μm; n = 3; **P* < 0.05, ***P* < 0.01.

**Figure S3.** Upregulation of MLK3 in T98G cells disrupts actin cytoskeleton rearrangement. Control, empty vector. Phalloidin was used to label F-actin (red), and DAPI was used to stain nuclei (blue). Scale bars, 50 μm.
