# Supplementary figures and images for: MLK3 Is Associated With Poor Prognosis in Patients With Glioblastomas and Actin Cytoskeleton Remodeling in Glioblastoma Cells
Source: Front Oncol. 2021 Feb 22;10:600762. doi: 10.3389/fonc.2020.600762 (PMC7937953; doi:10.3389/fonc.2020.600762)

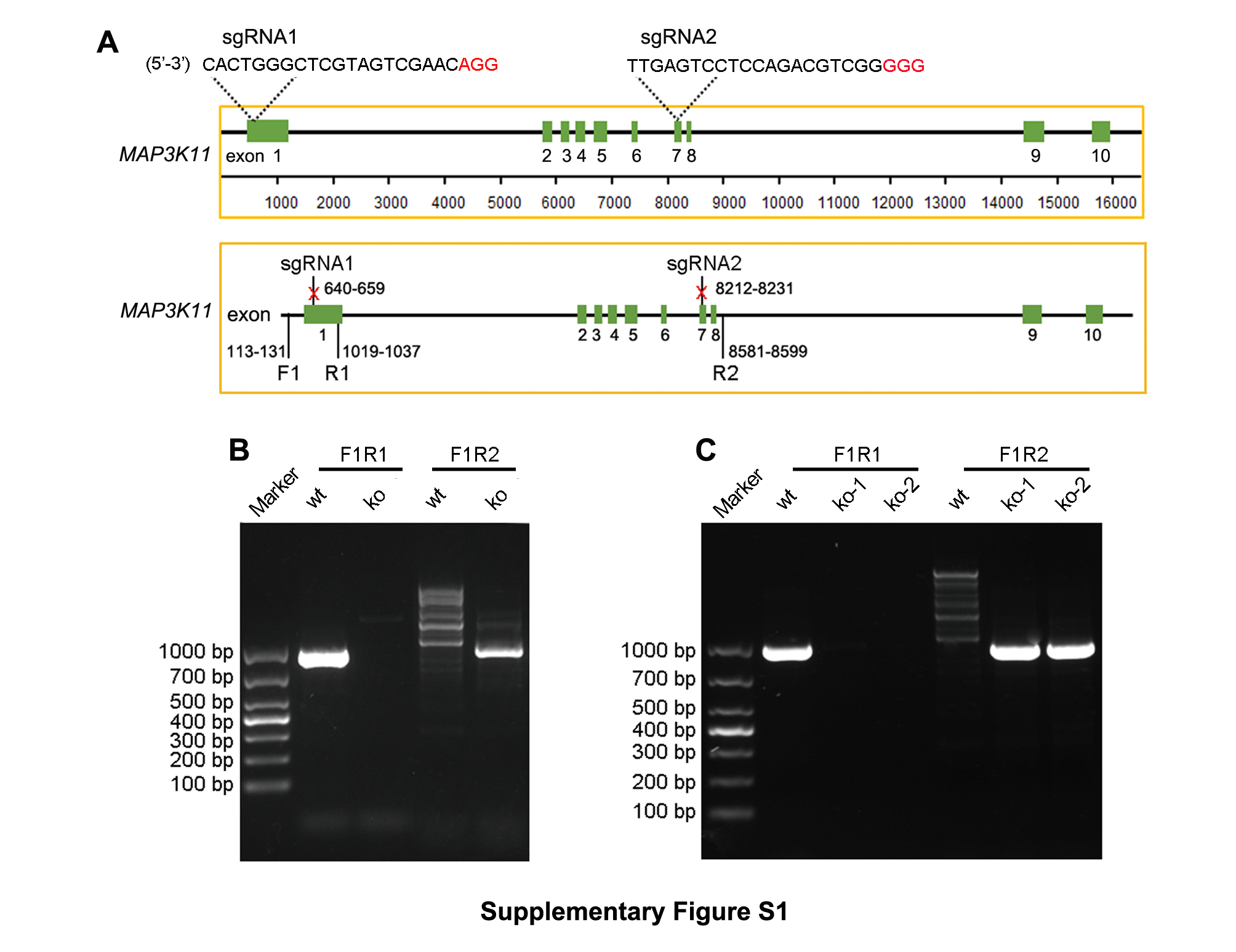

Supplement: Supplementary file 2 [file Image_1.tif]

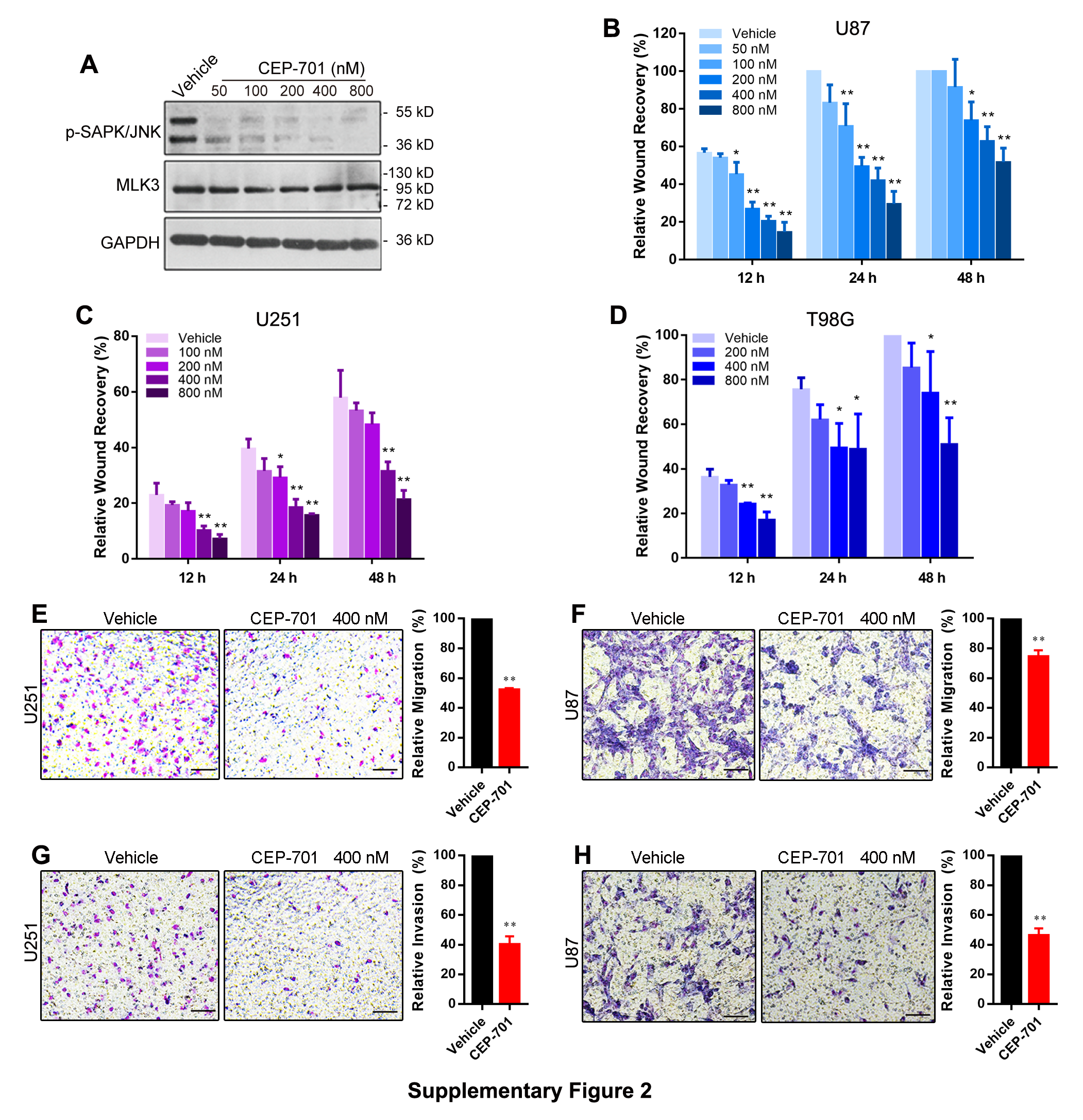

Supplement: Supplementary file 3 [file Image_2.tif]

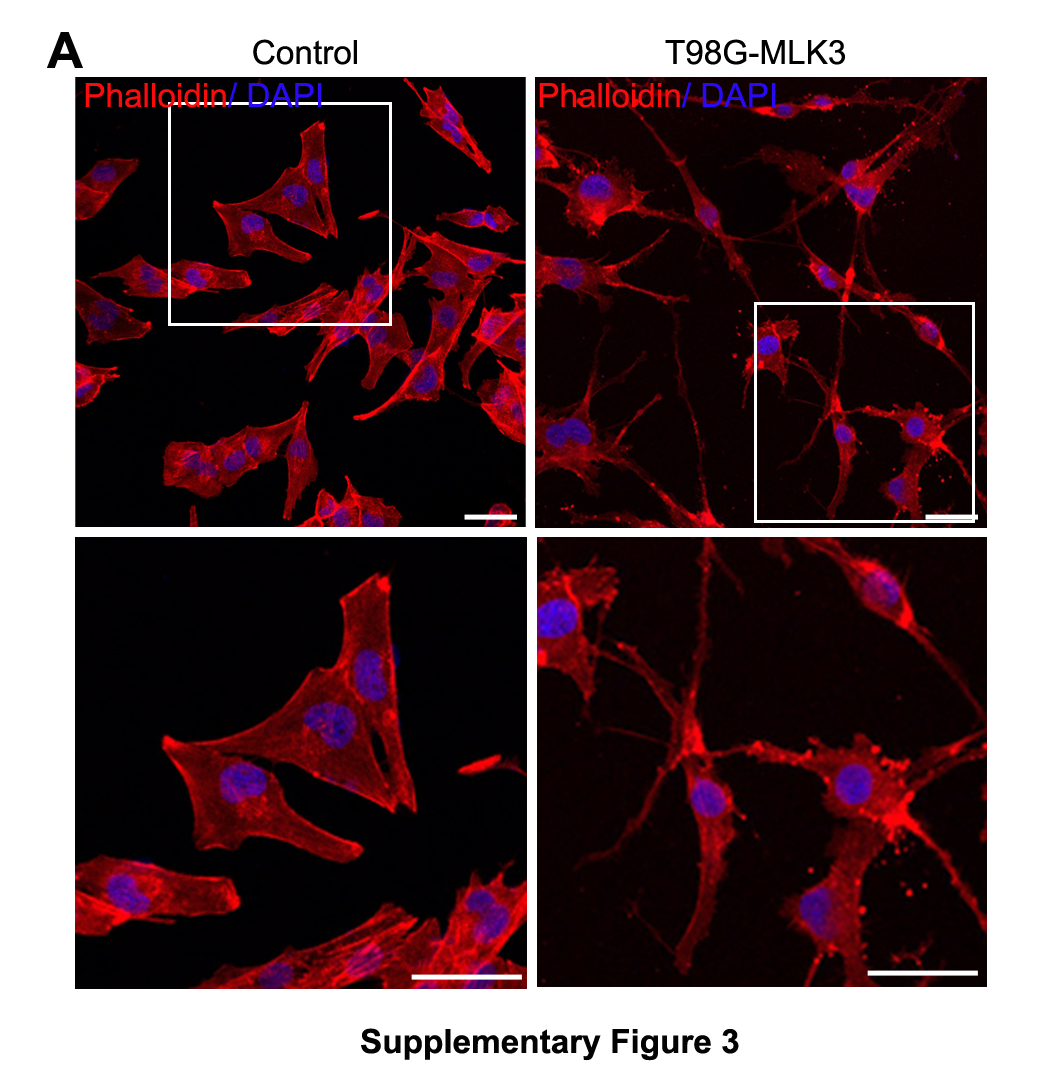

Supplement: Supplementary file 4 [file Image_3.tif]
